# MNPs-IHSPN nanoparticles in multi-application with absorption of bio drugs in vitro

**DOI:** 10.1016/j.bbrep.2021.101159

**Published:** 2021-10-23

**Authors:** Mansour Binandeh, Sadegh Rostamnia, Farrokh Karimi

**Affiliations:** a Faculty of Science, Department of Chemistry, University of Maragheh, Iran; b Faculty of Science, Department of Biotechnology and Environmental, University of Maragheh, Iran

**Keywords:** Magnetic nanoparticles, Silica-thiol coating, Imidazole protection, Cys, MTX

## Abstract

The aim of this project is to investigate the method of using a common buffer to determine the degree of stabilization and secretion of two drug molecules that have been analyzed in vitro. First, magnetic nanoparticles were synthesized and their structure was identified by instruments such as XPS (X-ray photoelectron spectroscopy) and FT-IR (Fourier transform infrared spectroscopy). The main purpose of this study was to investigate the stabilization and release of methotrexate on the surface of magnetic nanoparticles. The two temperatures were 37 and 25°, respectively. After reaction with the biomolecules, the adsorption rate for both drug molecules was about 60–80. PBS buffer was also used for diffusion of biomolecules and the results were analyzed by spectrophotometer analysis. With these results, the adsorption of cysteine and MTX was more than 60% and its release rate in MNPS-IHSPN was up to 90%, which means that high-strength stabilization and release by magnetic nanoparticles under external magnetic field and in vitro confirmed. The result of this project for the exchange of drugs by the surface of magnetic nanoparticles to repair damaged cells in the body of living organisms can be generalized.

## Graphical abstract

**Figure:**
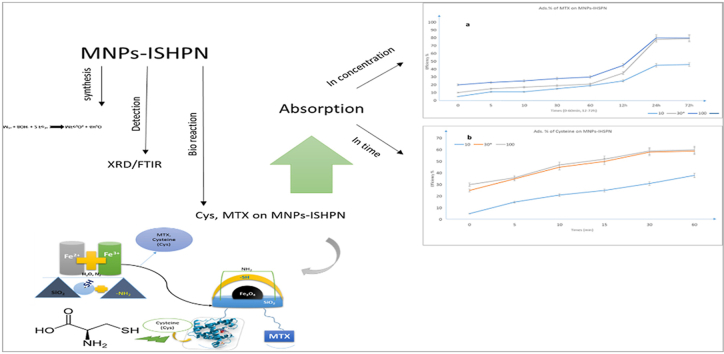

## 1 Introduction

In this regard, in nanoscience, a topic called magnetic nanoparticles has been developed and has provided one of the most important research fields for the stabilization and diffusion of various drug molecules. Therefore, in order to study the activity of magnetic nanoparticles, it is necessary to study the nanoparticles (ie their size), which are determined by laboratory studies with different analysis devices, which is suitable for biological metabolism between 5 and 100 nm. Thus, it can be concluded that if the size of nanoparticles is in this range, they can be used for various types of biomedical reactions, such as: to repair damaged cells by stabilizing and releasing biological drug molecules on the surface of magnetic nanoparticles, targeted transfer of Drug molecules to those cells [1].

### 1.1 Application and structure of magnetic nanoparticles

For a longer period of time, it has been tried to be assigned to nanoparticles with a magnetic approach and the most discussed topic is their surface. According to the previous experiment [9,10], uncoated magnetic nanoparticles can also stabilize and release protein, DNA. Therefore, for this purpose, different types of coatings have been used to increase the performance of magnetic nanoparticles, among which, silica coating (-SiOH) still has the first priority. In this study, another coating (mercapto, –SH) was used next to it to use the surface of nanoparticles for biomedical reactions (stabilization-release of various biochemical drugs) [[2], [3], [4], [5]].

### 1.2 Stabilization-release of methotrexate and cysteine by MNPs-IHSPN

**Fig. 1:**
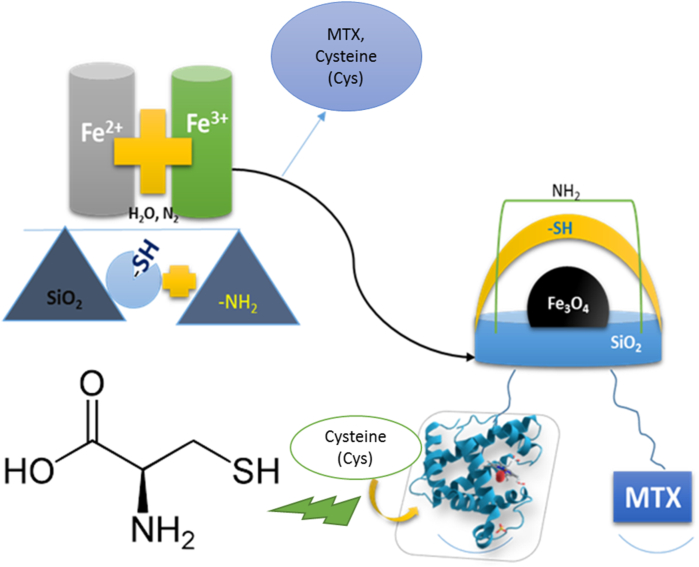
The general of MNPS-ISHPN formation and its reaction with biomedical biomolecules.

As explained, the applications of magnetic nanoparticles are many, including their very important use in the stabilization and release of important biopharmaceuticals such as methotrexate and cysteine. Methotrexate ((2S)-2-[(4-{[(2,4-Diaminopteridin-6-yl)methyl](methyl)amino}benzoyl)amino]pentanedioic acid) was first used to treat cancer and was given repeatedly during chemotherapy [6,7]. The drug enters the patient's body at a standard temperature of 37° and increases the body's resistance to cancer and helps macrophages to destroy damaged cells. In contrast, cysteine is an amino acid with the formula HO_2_CCH (NH_2_) CH_2_SH, which is used in the human body to combine various proteins. The appearance of this compound is white powder or crystals. This amino acid is not essential in the human diet and can be produced from the metabolism of compounds such as methionine. Cysteine is found in animal sources such as eggs, dairy and poultry, and plant sources such as onions, broccoli, and whole grains, as well as human and pig hair [[8], [9], [10]]. However, our goal in this project was to create suitable conditions for the stabilization and release of these two biopharmaceuticals in vitro. Stabilization of the protein drug cysteine was done for the first time in this project and methotrexate was also done for the first time with this nanocatalyst synthesized in this project, although it has been studied by many researchers and with many nanocatalysts [[11], [12], [13], [14], [15], [16], [17], [18], [19], [20], [21], [22], [23], [24], [25], [26]]. At 37 °C for methotrexate and 25 °C for cysteine, the two drugs were first stabilized separately on the surface of magnetic nanoparticles. Finally, they were completely separated from the surface of nanoparticles by buffer solution. The important point is that magnetic nanoparticles can be used as a suitable carrier without damage to these two drugs and can be easily separated from the reaction medium by an external magnetic field. If used in the human body, this separation without leaving a toxin It will be performed in vivo, which confirms the very good performance of magnetic nanoparticles (Fig. 1).

## 2 Methods

### 2.1 Materials

All solvents and chemicals are purchased from commercial Suppliers. Cysteine (C_3_H_7_NO_2_S, M; 121.16 g/mol) were obtained from Sigma (St. Louis, MO). Materials such as; ferrous chloride tetrahydrate (FeCl_2_·4H_2_O), ferric nitride Nona hydrate (Fe (NO_3_)_3_·9H_2_O) and sodium hydroxide (NaOH) were purchased from Merck KGaA (Darmstadt, Germany). And, phosphate buffered saline (PBS (pHs 6.0–8.0)), argon gas, HCl, methanol, TritonX100, EDTA, Boric acid, NaCl, glutaraldehyde and Salmon sperm (protein) sodium salt is purchased from Sino-pharm Chemical Reagent Co. (Shanghai, China). Methotrexate (Ibo, Canada) with a molecular weight (454.44 g/mol, NHS (*n*-hydroxysuccinimide) molecular weight (115.09 ppm), EDC (1-ethyl-3- (3- (dimethyl)-amino) -propylene (carbon dioxide) with molecular weight (155.25 g/mol), imidazole (C_3_N_2_H_4_, molar mass 68.077 g/mol), (3-choloropropyl) triethoxysilane or CPTES, (molecular weight 198.72, 97% purity), (3-mercaptopropyl) triethoxysilane or MPTES, (molecular weight 196.34 g/mol, 95% purity), APTES (3-amino-propyl tri-methoxysilane), DMSO (dimethyl sulfoxide), sodium hydroxide and chloride-containing acid with a concentration of 0.1 M, TEOS (tetraethyl-orthosilicate), Hydrazine (34% by weight aqueous solution, reducer) were purchased from Sigma-Aldrich Co. (St Louis, MO, USA). Protein used in the lab is models (Maragheh, Iran). Deionized water was used in each experiment.

### 2.2 Synthesis of Fe_3_O_4_@SIO_2_/SH/NH_2_ nanoparticles

Chemical Co-precipitation is simple method to synthesize also of magnetic nanoparticles with core/shell structure [[27], [28], [29], [30]]. So according to this method first, sample container iron salts with amounts of 1–2 (1.5 mg of FeCl_2_·4H_2_O and 3 mg of Fe (NO_3_)_3_·9H_2_O) were dissolved in distilled water. The reaction temperature was 25 °C and high-intensity spinning under inert nitrogen gas. Because the nanoparticle substrate can be severely oxidized in the vicinity of ambient air, therefore, after 3 h, about 3 ml of tetraethyl orthosilicate is used, which in addition to a suitable coating for magnetic nanoparticles to prevent them from oxidizing, also increases their efficiency in biological applications.

After the synthesis of Fe_3_O_4_@SiO_2_/SH magnetic nanoparticles, 100 mg of the same magnetic nanoparticles were weighed and dissolved in 4 ml of distilled water to dissolve completely in water. In the other part, 3 ml of APTES solution (3-aminopropyl 3-methoxy silane) and then magnetic nanoparticles Fe_3_O_4_@SiO_2_ were prepared, double distilled water 4 ml until completely dissolved in water twice. After complete dissolution of the sample, it was placed under reflex conditions at 100 °C and 250 μl of binder (CPTES) was added to the solution. Then, for 24 h, 250 μl (MPTES) was added to the solution. Within another 36 h, 250 μl of *N*-methyl imidazole was added to remove chloride from the first binder. In the case of a heater, these solutions are completely dissolved and supplied through a magnet. The solution is completely dissolved in magnetic nanoparticles drop by drop in APTES solution and placed overnight in non-temperature conditions (room temperature, 25 °C). Finally, we place the Fe_3_O_4_@SiO_2_/SH/NH_2_ magnetic nanoparticles prepared by distilled water, ionized twice, and placed in an oven to dry. Finally, magnetic nanoparticles containing a yellowish brown amine functional group were prepared.

### 2.3 Magnetic Fe_3_O_4_@SiO_2_/SH/NH_2_ nanoparticle for stabilization of MTX drug molecule

The concentration of 25 mg of nanoparticles (dissolved in water) in 100 μl of methotrexate (at 372 nm), in a dimethyl sulfoxide solvent and a reaction temperature of 37 °C under vigorous stirring (300 at a speed of rotation per second) for 36hr of times. The synthesis was carried out under conditions where the magnetic nanoparticles were completely dissolved in the water solvent to distribute the particles uniformly; in another test tube, the methotrexate particles were dissolved in a solution of dimethyl sulfoxide and the reason for using this solution (dimethyl sulfoxide) was therefore to reduce the solubility of methotrexate in water. When methotrexate was conjugated with magnetic nanoparticles, two materials: (1-ethyl-3- (3- (dimethylamino) propyl) carbonyl amide (7.5 μl)) and *N*-hydroxy succinimide (15 μl)). Both of them, as we have mentioned, play an important role in the process of stabilizing methotrexate at the surface of the nanoparticles and creating a link between the two. Finally, after completion of the reaction, the nanoparticles are detached by a magnetic magnet and the prepared solution is prepared from the samples with a mixed mass (consisting of nanoparticles and conjugated molecules conjugated to their surface) for absorption analysis, which is then thoroughly investigated.

### 2.4 Cysteine adsorption of aqueous solution

Cysteine adsorption experiments were carried out in batch-wise. Approximately 25 mg of magnetic silica nanoparticles were mixed with 1 ml of various concentrations of Cys solution in water. The mixture was shaken at room temperature for 1 h, which proved to be sufficient period to reach equilibrium. Then the magnetic particles were separated with help of the permanent magnet and the supernatant was assayed for remaining protein concentration by the UV–Vis spectrophotometer at 318 nm. The adsorbed amount of protein was calculated by mass balance.

## 3 Results

In this section, we will first try to prepare the above magnetic nanocomposite under the core/shell structure and then use it by highly specialized analyzes such as SEM XPS, FT-IR to detect the MNPS-IHSPN structure.

### 3.1 SEM spectrum of Fe_3_O_4_@SiO_2_/SH

**Fig. 2:**
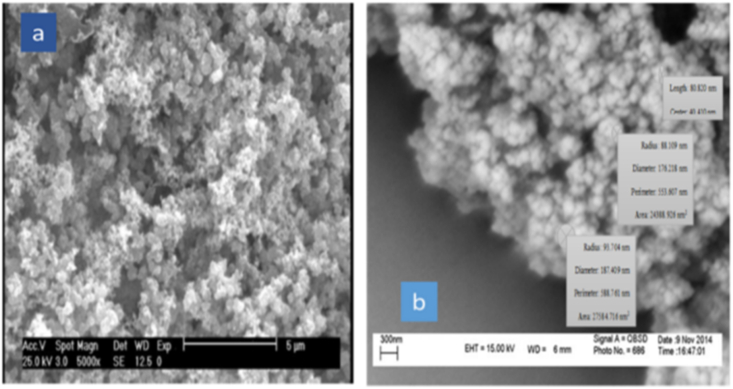
a) Fe_3_O_4_@SiO_2_/SH/NH_2_, b) Fe_3_O_4_@SiO_2_ SEM analyses of nanoparticles.

SEM is an analysis to observe the surface of magnetic nanoparticles, which is actually an image of their core/shell structure, measured on the basis of nanometer data. What is very important is that with the increase of silica-thiol coating and functional groups such as amine-imidazole on the surface of nanoparticles, their structure has become more regular, which can be clearly seen in Fig. 2(a and b).

### 3.2 XPS spectrum of Fe_3_O_4_@SiO_2_/SH

**Fig. 3:**
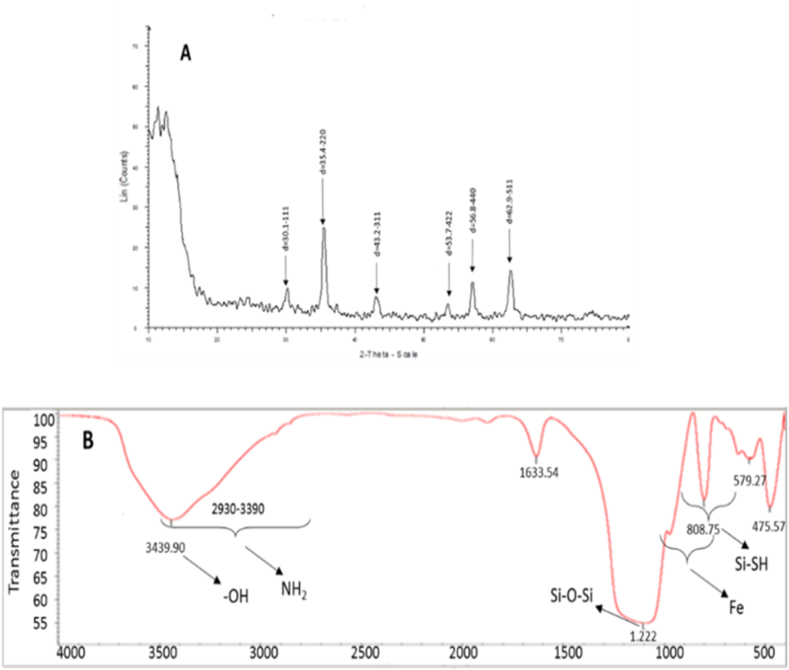
Pictures are A) XPS, B) FT-IR of Fe_3_O_4_@SiO_2_/SH/NH_2_ magnetic naoparticles

XPS analysis, the X-ray diffraction, is an analysis that shows the number of electron transmissions using X-rays at energy levels in orbital layers and electron capacities. Fe_3_O_4_ pixels are similar to 3 theta pixels, and in pixels between the processing of 30.1 and 35.4, 43.2, 53.7, 56.9, 62.9 for Fe–O–Si and 220, 311 for –SH, 422, 511, 440 cm^−1^ for –NH is visible in the (Fig. 3A). Results indicate that Fe_3_O_4_ was synthesized and no changes were made in its crystalline structure. Two new couriers were observed in 3 thetas of 40, 46.7 and 62.9 cm^−1^, which are similar to the peaks of 111, 200 and 422 cm^−1^, which confirm the completion of –SH/-NH groups [**8**] to the surface of the magnetite magnetic nanoparticles with a silica-mercapto substrate and factorized with the functional imidazole hetro-polymer and amine linker.

### 3.3 FT-IR spectrum of Fe_3_O_4_@SiO_2_/SH/NH_2_ (MNPS-IHSPN)

In order to show the bond between the functional groups to make the covalent bond, FT-IR analysis is used, the range of frequencies is 500–4000 cm^−1^, the absorption band for the 808 SiO_2_ functional group and 1.222 cm^−1^ Si–O–Si. In the present magnetic nanocatalysis, the amount of silica coating at 879 and 694 and for mercapto (-SH functional group) was at 1.212 cm^−1^ and so, 2930-3390 cm^−1^ is about amino linker (-NH_2_ functional group, which after the addition of sulfur functional group, the peak rate became narrower ie more regular. This would indicate an increase in the level of performance of the magnetic nanoparticles (Fig. 3B).

### 3.4 Results of protein and MTX loaded onto magnetic nanoparticles Fe_3_O_4_@SiO_2_/SH/NH_2_) by spectrophotometry

The purpose of this section is to investigate the adsorption of drug biomolecules in the substrate of magnetic nanoparticles. Therefore, the absorption rate can be investigated using Equation [**27**–**30**]. Under standard conditions, 30 μg per microliter of protein and biomolecule is dissolved in 2 ml of sterile water and then twice 25 mg of magnetic nanoparticles are weighed in two separate containers on each of the biomolecules, protein (60 min)., 30 μg/ml (318 nm) and drug (36 h, 10 μg/ml, 372 nm) were dissolved separately.

#### 3.4.1 For MTX

After methotrexate has been stabilized on a surface of Fe_3_O_4_@SiO_2_/SH/NH_2_ magnetic nanoparticles with different methods, we will now consider the methotrexate release rate. This is done with a phosphate buffer solution which is a mixture of 4 K_2_HPO_3_, Na_2_HPO_3_, KCl, and NaCl salts in a specified amount of distilled water, which is ready for use in the reaction after the autoclave. Using phosphate buffered saline, sodium hydroxide, and chloride acid adjust the pH to 8.8 to adjust the pH of phosphate buffer of methotrexate on the surface of nanoparticles and with sodium hydroxide and chloride. And the rate of release has been measured over a period of 2 h–72 h at pH of 7.28–8.0. Looking at the data in the chart, it can be concluded that after 24 h, the release of the methotrexate drug molecule from the surface of nanoparticles is about 80% at pH 7.8. With these results, can be said that is a full stabilization of MTX drug molecule on MNPs-IHSPN in vitro. The results showed that the absorption rate of drug on the nanoparticles were about 80%.

#### 3.4.2 For protein

**Fig. 4:**
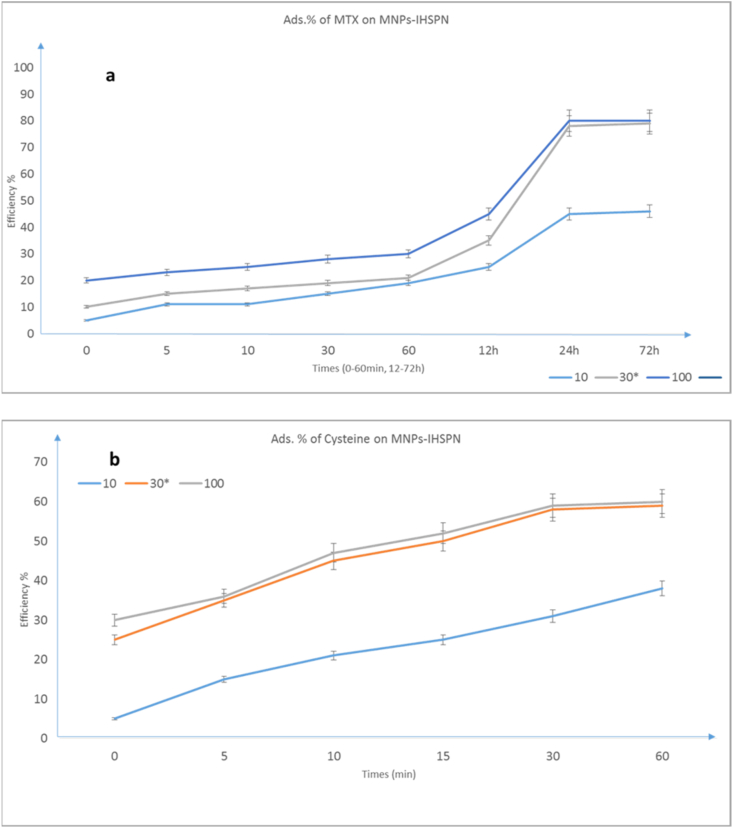
Results of absorbed MTX (12–72 h, 10 μg/ml) (a), Cys (0–60 min, 30 μg/ml) (b) biomolecules on MNPs-IHSPN nanoparticles.

This reaction was performed at room temperature and in distilled water. Sampling was performed at 0–60 min and stored in separate containers. Each sample then reached a volume of 200 μl in the buffer solution (the buffer solution was used to separate the protein from the surface of the magnetic nanoparticles to evaluate the amount of protein adsorbed relative to the solution in which the protein was purely dissolved) and inside the machine blocks. Spectrophotometry was analyzed. At a wavelength of 318 nm, the absorption rate of each sample was taken and the results showed that the adsorption rate of protein on magnetic nanoparticles is >60%. The results are shown in (Fig. 4 (a)MTX, b)Cys). All Error bars are selected as standard, indicating the accuracy of the data presented in the chart.

## 4 Discussion

### 4.1 Stabilization of methotrexate and protein to the surface of the magnetic nanoparticles Fe_3_O_4_@SiO_2_/SH/NH_2,_ EDX analysis for Cys, MTX

**Fig. 5:**
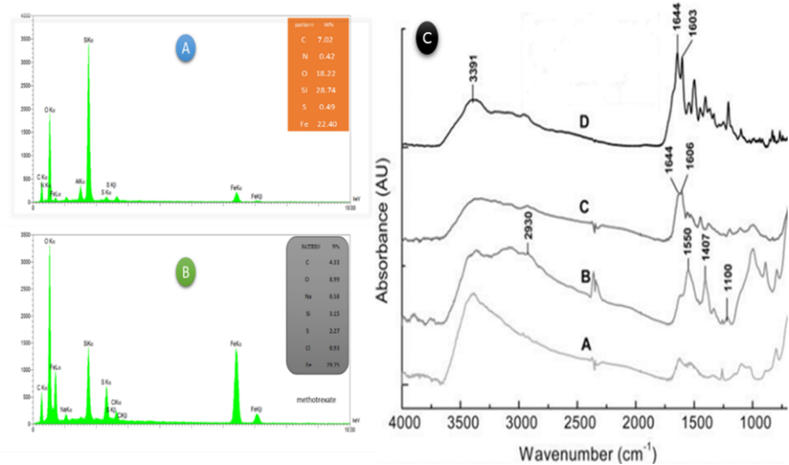
Results of EDX analysis. Fig. 5A is for Cys protein and, Fig. 5B for MTX biodrug. And Fig. 5C, result of FT-IR analysis for absorption of MTX biodrug on MNPs-IHSPN nanoparticles, A) Fe_3_O_4_@SiO_2_, B) Fe_3_O_4_@SiO_2_/SH_,_ amine linker consisting of 2930 cm^−1^ ppm for the (-CH) group, 1407 and 1550 cm^−1^, for the –NH_2_ group and 1100 cm^−1^ for the Si–O group and furthermore so, 1112 cm^−1^ is for –SH group, C) Fe_3_O_4_@SiO_2_/SH/NH_2_, SH-NH_2_ linker and fixed methotrexate on it includes 1606 and 1644 pixels cm^−1^ for covalent bond between carboxyl group (-COOH) methotrexate and amine group (-NH_2_), and D) Fe_3_O_4_@SiO_2_/SH/NH_2_/MTX, methotrexate structure including peak 3391 cm^−1^ related the group (-OH) and peaks of and 1603 and 1644 cm^−1^ correspond to poor absorption of methotrexate on surface of an amine-bonded magnetic nanoparticle.

In this discussion, the atomic weight percentages of the elements in each of the MTX and protein reactors and Fe_3_O_4_@SiO_2_/SH/NH_2_ were measured, and the relationship between these molecules can be determined by the weight percentages. EDX analysis showed that both reagents were linked together in the product. Also, the elements Fekα and Fekβ are shown with the elements Si (with a strong peak) and O in the product Fe_3_O_4_@SiO_2_. The results of EDX analysis show that the connection of factor N at 750 keV and factor O at 1100 keV and factor N at 750 keV and factor C at 600 keV because they are in the same line, so it may be said that this method is electrostatic. Same for NO and covalent bond for CN. Element O in the functional group O_2_ is coated silica (SiO_2_) and element N is the NH_2_ agent. Evidence of secondary EDX analysis is the spectrophotometer for this tissue. The result of adsorption and correlation between magnetic nanoparticles and MTX protein (Fig. 5B) or Cys (Fig. 5A) can be seen using EDX analysis (Fig. 5).

#### 4.1.1 FT-IR analysis for MTX

In the FT-IR spectrum (Fig. 5C), the nanoparticles with silica coating, we observe no change in the structure of the nanocomposite of magnetism. By examining the pixels of the four samples, the following results were obtained: A) the structure of magnetic nanoparticles with coating silica, B) the structure of magnetic nanoparticles with amine linker consisting of 2930 cm^−1^ ppm for the (-CH) group, 1407 and 1550 cm^−1^, for the –NH_2_ group and 1100 cm^−1^ for the Si–O group and furthermore so, 1112 cm^−1^ is for –SH group, C) Magnetic nanoparticle structure with SH-NH_2_ linker and fixed methotrexate on it includes 1606 and 1644 pixels cm^−1^ for covalent bond between carboxyl group (-COOH) methotrexate and amine group (-NH_2_), and D) methotrexate structure including peak 3391 cm^−1^ related the group (-OH) and peaks of and 1603 and 1644 cm^−1^ correspond to poor absorption of methotrexate on surface of an amine-bonded magnetic nanoparticle. –SH linker present in the ionic complex in the structure of the magnetic nanoparticles improves the covalent bond between the amine linker of the nanoparticles with the carboxyl linker of the methotrexate bio-drug and is a sharp and regular peak that shows drug absorption and strong bonding with the nanoparticles. So it can be said that the presence of ionic liquid composition increases the rate of drug absorption on the surface of the nanoparticles.

### 4.2 Stability of magnetic nanoparticles in repeated use after recycling

**Fig. 6:**
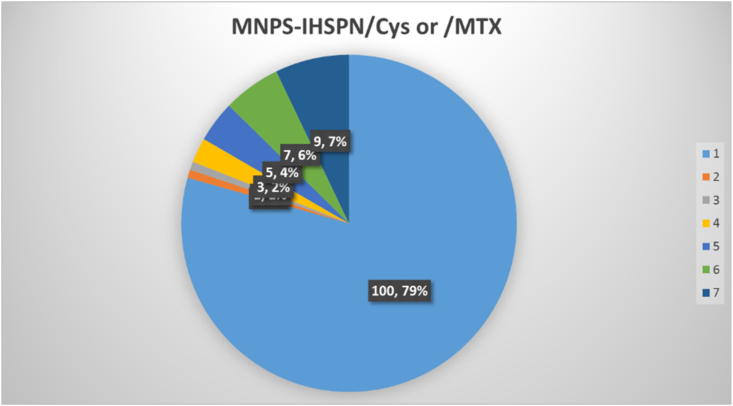
Efficiency of MNPs-IHSPN nanoparticles in stabilization of MTX, Cys biomolecules for repeated use over a period of 7 days.

The results of the magnetic nanoparticles with silica coating for MTX or Cys adsorptions were analyzed by spectrophotometric analysis over a period of 12–170 h for 7 periods, and results showed that the efficiency of nanoparticles in the application re-use to stabilization of biomolecules, even decreased by 5% over the course of 10%. Magnetic nanoparticles are very important for sustainability under favorable reaction conditions and having the ability to re-use of them. Magnitude of this stability has been investigated in 7 days (the reaction process and optimal conditions are the same as in the discussion and conclusion). The results are shown in (Fig. 6).

### 4.3 Results of the absorption of protein by electrophoresis

**Fig. 7:**
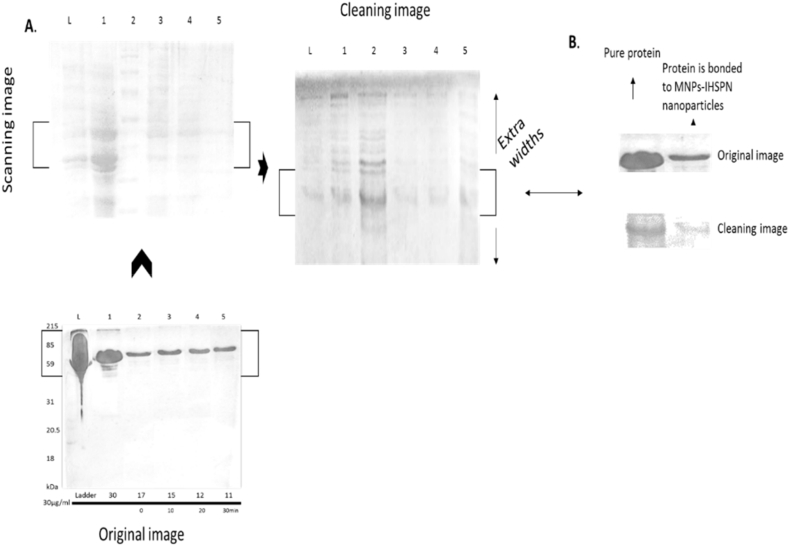
A. **The overall width of the stain contrast in gel electrophoresis**. It is clear from the figure that from the bottom to the top and then to the right, the scan of the spots has started and the light width of the spots has decreased from 2 to 3 halves. The maximum contrast brightness was observed in 4–5 spots (ie, a sample taken at 20 and 30 min). The results of the protein adsorption test on the surface of the magnetic nanoparticles show a perfect agreement with the width of the spots. B. **Accurate detection of the brightness difference of the spots in the gel by electrophoresis**. By comparing two important spots (the first related to nanoparticle unbonded protein and the second to nanoparticle bonded) at the beginning of the transient spot with the end of the electrophoresis scan, when the brightness of the spots contrast in the moment (when the protein is on magnetic nanoparticles It is at zero and the reaction has not yet taken place (reduced by about half) and this indicates that the protein is absorbed in the first place.

In this section, proteins are analyzed by electrophoresis analysis. Electrophoresis analysis is based on adsorption at the time of adsorption. Here, vertical electrophoresis is used to measure protein uptake. According to (Fig. 7), it can be seen that the amount of stained samples in the range of 0–60 min, due to the adsorption of protein on the surface of magnetic nanoparticles gradually fades, which this fading stain indicates that the molecules Bio-adsorbed on the surface of magnetic nanoparticles. Therefore, spectrophotometric adsorption and electrophoresis of both devices showed acceptable results for this adsorption. First, in 5 different patterns for protein in three separate experiments at a dose of 30 μg/ml and at 0–60 min on a sol-gel plate (different solutions of supernatants and biomolecules (proteins) -MNPs-IHSPN, and Finally, the last point of mass of magnetic nanoparticles containing biomolecules). In the first line, the ladder is the first point, the second line is protein staining without nanoparticles (sample 1), the third line contains any protein mixed in nanoparticles (without catalyst) at time zero (sample 2), the fourth line contains fixed protein in nanoparticles At 5 min (sample 3), the fifth line is at 15 min for protein (sample 4) and the sixth line is for 30 min for protein (Example 5). The sol-gel was then placed on electrophoresis and began scanning and staining. After 2–5 h, the results were observed in the same stains of about **60%** of the apparently weaker stains, apart from samples containing the net amount of protein, and staining showed that the nanoparticle surface adsorption rate was at least 60%, which is the result of adsorption The protein is on the surface of the magnetic nanoparticles, which confirms the stability of the biological molecules (proteins) in the nanoparticles, which the data in (Table 1 (pictured below), and the results of electrophoresis analysis in Fig. 7 (**A**, **B**)) and (Image above) is visible.

**Table 1:** The data obtained is categorized in Table 1.

Pattern 100 (μl/ml) Times (minutes)	Protein (unadsorbed) Adsorption (μg/ml)	protein-MNPS-IHSPN Adsorption (μg/ml), %
	0.489	1.09
0	**0.285**	**0.59 … 55%**
5 min	**0.225**	**0.21 … 60%**
10 min	**0.215**	**0.23 … 65%**
30 min	**0.215**	**0.21 … 65%**

## 5 Conclusion

In this project, the synthesis of magnetic nanoparticles with silica-thiol coating has a very important goal, ie to absorb, stabilize and release drug molecules (methotrexate and cysteine) under laboratory conditions. Therefore, according to previous results [**27**–**30**], the use of magnetic nanoparticles with new coatings increases the adsorption percentage of drug biomolecules. Even with increasing coatings, the performance of magnetic nanoparticles becomes better and more convenient. We use these results to create a surface beyond the adsorption and stabilization of biopharmaceuticals on the surface of magnetic nanoparticles by using the target biopharmaceutical by targeted transfer by an external magnetic field to enter the body to repair damaged cells.

## Ethics approval and consent to participate

All of co-authors do it.

## Consent for publication

All of co-authors and Maragheh University do it.

## Availability of data and materials

All of data and materials are done.

## Funding

No.

## Author's contributions

Not applicable.

## Author's information (optional)

Dr. Binandeh is researcher in organic-med chemistry field. Prof. Rostamnia and Dr. Karimi are supervisor for this paper.

## Declaration of competing interest

Any co-authors not have with together.
